# Bioinformatics analysis of various signal peptides for periplasmic expression of parathyroid hormone in *E.coli*

**DOI:** 10.25122/jml-2018-0049

**Published:** 2019

**Authors:** Aref Doozandeh Juibari, Sina Ramezani, Mohammad Hosein Rezadoust

**Affiliations:** 1. Department of Biology, University of Guilan, Rasht, Iran; 2. Faculty of Agriculture, University of Guilan, Rasht, Iran

**Keywords:** Parathyroid hormone (PTH), periplasmic expression, pharmaceutical, in silico, *E.coli*

## Abstract

Hypoparathyroidism is a rare endocrine disease which is characterized by the deficiency of serum calcium levels. RhPTH is prescribed as a therapy for the management of refractory hypoparathyroidism. The aim of this study is to investigate 32 signal peptides of gram-negative bacterial origin and evaluate their potential for efficient secretion of recombinant human PTH (1–84)In E.coli to obtain higher expression of recombinant PTH in bacterial systems by using this fusion partner. SignalP and ProtParam servers were employed to predict the presence and location of signal peptide cleavage sites in protein sequence and computation of various physical and chemical parameters of protein respectively. Also, SOLpro server was applied for prediction of the protein solubility. Then ProtComp and SecretomeP online servers were employed to determine protein location. The evaluations showed that theoretically two signal peptides Lipopolysaccharide export system protein LptA (lptA) and Periplasmic pH-dependent serine endoprotease DegQ (degQ) are the most appropriate signal peptides examined. Due to the lack of post-translational modification in PTH, its periplasmic expression has preferences. Based on the results of this study, using bioinformatics and reliable servers signal peptides with appropriate secretory potential can be obtained which lead to the highest expression level.

## Introduction

Parathyroid hormone (PTH) is an endocrine hormone, which is secreted by the chief cells of the parathyroid glands that is essential in bone remodeling. PTH is secreted in response to low blood serum calcium levels in the bloodstream. Parathyroid hormone indirectly increases bone resorption through osteoclast activity within the human bone marrow, which causes it to release more ionic calcium into the blood to elevate serum calcium levels. The production of 1,25(OH)_2_D in the kidney is tightly controlled, increased by parathyroid hormone. PTH is a polypeptide containing 84 amino acids and its half-life is estimated to be 4 minutes. Moreover, it has a molecular mass of almost 9.5 kDa.

PTH is a single-stranded polypeptide chain that is free of disulfide bonding and glycosylation sites. Hypoparathyroidism is a rare endocrine disease which is characterized by hypocalcaemia because of inappropriately low or absent levels of parathyroid hormone [1, 2]. Hypoparathyroidism is evaluated to occur after almost 0.5–6.6% of total thyroidectomies. The condition results from a surgical procedure in about 75% of patients, caused by the unavoidable or accidental removal of or damage to the parathyroid glands during various head and neck operations in adults. Moreover, it can be due to congenital, autoimmune, or caused by various other factors [3, 4]. Hypocalcemia can present dramatically as altered mental status, stridor, tetany, seizures or refractory congestive heart failure [5]. In January 2015, the FDA approved a bioengineered recombinant human PTH(1–84) [rhPTH(1–84)], as an adjunct to calcium and vitamin D therapy for the management of refractory hypoparathyroidism[6].

RhPTH, a biologically active 84-amino acid protein effects directly on the bones and kidneys and indirectly on the intestines to balance calcium and phosphate homeostasis [7].

*Escherichia coli(E.coli)* is one of the organisms of option for the production of recombinant proteins, since these cells can be easily manipulated, are cultured cheaply and grow rapidly.

The commercial success of any recombinant protein depends on its potential for large scale production and lower costs of the final product. Thus the cost of production of a recombinant product can be reduced by enhancing the efficiency of large scale production [8].

Overexpression of lots of proteins in *E.coli* host results in a mass of insoluble protein as inclusion bodies, where the efficiency of production of active proteins is very low, biologically, and purification methods are often expensive and laborious. A replacement method of preventing the formation of inclusion bodies is secretion of proteins to bacterial periplasmic space using appropriate signal peptides [9, 10]. By directing the protein of interest into the periplasmic space, the following processes of protein separation become facilitated, thereby reducing the downstream costs. In bacteria, translocation of proteins across the cytoplasmic membrane towards the periplasmic space is usually mediated by the interaction of their N-terminal signal peptides with the protein secretion machinery. In order to obtain a higher output from the recombinant protein of interest, one can add signal peptide to the protein of interest, artificially. Immediately after translocation, signal peptides are proteolytically removed by signal peptidase at a very specific site.

Signal peptides have three main functional and structural regions, counting N-base terminal region, hydrophobic region or h, and highly polarized C-terminal region. Recognition of signal peptides and correct cutting sites are extremely important for obtaining a proper function of the protein. To transfer proteins from cytosols into the periplasmic space , *E.coli* has different systems [11]. Transference from the cytoplasm to periplasm occurs through arginine hybrid transportation pathway (Tat) or sec-dependent pathway [12].

The aim of this study is to investigate 32 signal peptides with a gram-negative bacterial origin and evaluate their potential for efficient secretion of recombinant human PTH(1–84) in *E.coli* to obtain higher expression of recombinant PTH in bacterial systems by using this fusion partner.

## Material and Methods

### Investigation of signal peptides sequence

The amino acid sequence of 32 signal peptides with a gram-negative bacterial origin was obtained from UniProtKB database from ExPASy server. The collected sequences were used to achieve suitable signal peptides to enhance the value and accuracy of periplasmic expression of recombinant PTH in *E.coli*.

### Prediction for the location of the cleavage site of the signal peptides

SignalPserver 4.1(http://www.cbs.dtu.dk/services/SignalP/) was applied to predict the presence and location of signal peptide cleavage sites in protein sequence. The accuracy in detecting signal peptides has been estimated to be around 87% [13]. SignalP server 3 was employed to study the three regions of n, h, and c of signal peptides.

### Computation of physiochemical characteristics of signal peptides

Protparam (http://web.expasy.org/protparam/) is a tool which allows the computation of various physical and chemical parameters for entered protein sequence. The computed parameters include the molecular weight, theoretical pI, estimated half-life, instability index, aliphatic index and grand average of hydropathicity (GRAVY), so protparam was employed for evaluation of different physiochemical characteristics [14].

### Prediction of protein solubility

SOLpro server (http://scratch.proteomics.ics.uci.edu/) is an accurate sequence-based prediction of the protein solubility with an overall accuracy of over 74%, was used to predict protein solubility after overexpression in *E.coli* [15, 16].

### Prediction of secreted protein location

Two servers were used in the investigation of protein location after secretion. Protcomp server 9 (http://www.softberry.com/berry) and SecretomeP 2.0 (http://www.cbs.dtu.dk/services/SecretomeP) used lots of different servers to determine protein location based on protein characteristics [13, 17].

## Results

### Isolation of signal peptides

In order to find signal peptides with secretion ability and other desirable features, the amino acid sequence of 32 secretory signal peptides with a gram-negative bacterial origin was investigated. The sequences with confirmed secretion potential, after extraction from the UniProt database (UniProtKB) linked to the parathyroid hormone sequence, were compared in several stages to obtain more secretion ability of PTH in *E. coli* (Table 1).

**Table 1:** 

No	Signal peptides	N-region	H-region	C-region	Cleavage site	D-score
1	appA	1–4(4)	5–16(12)	17–22(6)	AFA-MS	0.787
2	dsbD	1–4(4)	5–13(9)	14–19(6)	VFA-MS	0.625
3	degQ	1–6(6)	7–21(15)	22–27(6)	AVA-MS	0.742
4	cueO	1–9(9)	10–21(12)	22–28(7)	No	0.367
5	pldA	1–5(5)	6–14(9)	15–20(6)	No	0.302
6	ushA	1–6(6)	7–18(12)	19–25(7)	ALA-MS	0.726
7	pagP	1–6(6)	7–18(12)	19–25(7)	No	0.217
8	fhuA	1–15(15)	16–26(11)	27–33(7)	No	0.435
9	ptrA	1–8(8)	9–17(9)	18–23(6)	SQA-MS	0.886
10	lptA	1–11(11)	12–21(10)	22–27(6)	AFA-MS	0.899
11	bepA	1–8(8)	9–20(12)	21–27(7)	AFA-MS	0.759
12	dacC	1–9(9)	10–20(11)	21–27(7)	No	0.501
13	agp	1–4(4)	5–16(12)	17–22(6)	AQA-MS	0.867
14	malS	1–3(3)	4–11(8)	12–17(6)	AVA-MS	0.805
15	spy	1–5(5)	6–17(12)	18–23(6)	AHA-MS	0.776
16	amiC	1–15(15)	16–25(10)	26–31(6)	No	0.305
17	tolB	1–6(6)	7–15(9)	16–21(6)	No	0.403
18	rseB	1–6(6)	7–16(10)	17–23(7)	ASA-MS	0.817
19	amtB	1–7(7)	8–16(9)	17–22(6)	No	0.457
20	dsbC	1–4(4)	5–13(9)	14–20(7)	AQA-MS	0.850
21	amiA	1–18(18)	19–27(9)	28–34(7)	AIA-MS	0.661
22	torZ	1–12(12)	13–24(12)	25–31(7)	No	0.335
23	fhuD	1–11(11)	12–23(12)	24–30(7)	AHA-MS	0.725
24	zinT	1–7(7)	8–17(10)	18–23(6)	No	0.484
25	mglB	1–4(4)	5–16(12)	17–23(7)	AHA-MS	0.860
26	eco	1–4(4)	5–14(10)	15–20(6)	AWA-MS	0.874
27	nikA	1–7(7)	8–16(9)	17–22(6)	VHA-MS	0.621
28	mepH	1–9(9)	10–21(12)	22–27(6)	AHA-MS	0.788
29	fadL	1–9(9)	10–19(10)	20–25(6)	AWS-MS	0.773
30	ompL	1–5(5)	6–14(9)	15–20(6)	VFA-MS	0.850
31	ompN	1–5(5)	6–14(9)	15–21(7)	AHA-MS	0.900
32	thiB	1–4(4)	5–12(8)	13–18(6)	VFA-MS	0.792

### Prediction of signal peptides cleavage site

Appropriate signal peptides linked to PTH in *E. coli* were predicted by SignalP-4.1, one of the commonly used and reliable servers for the prediction of the signal peptides cleavage site. Signal peptides with the ability to cleave at the cut-off site can be detected, based on the results of this server. The potential signal peptide was predicted using D-score discriminating score, termed D-score with default cutoff value of 0.5 is used as a basis for predicting the cleavage potential of sequences (Table 2).

**Table 2:** 

No.	Signal peptides	Amino acid length	MW	PI	GRAVY	Aliphatic index	Instability	Solubility	Solubility (%)
1	appA	22	11922.89	9.40	–0.275	100.28	47.08	INSOLUBLE	0.67923
2	dsbD	19	11665.54	9.25	–0.282	97.50	41.96	INSOLUBLE	0.59127
3	degQ	27	12315.22	9.57	–0.346	95.80	37.54	INSOLUBLE	0.63859
4	ushA	25	12171.09	9.40	–0.32	98.45	41.63	INSOLUBLE	0.57392
5	ptrA	23	12151.11	9.63	–0.376	95.74	46.87	INSOLUBLE	0.68441
6	lptA	27	12387.37	9.70	–0.326	96.70	38.71	INSOLUBLE	0.63528
7	bepA	27	12442.47	9.75	–0.335	93.21	40.32	INSOLUBLE	0.61099
8	agp	22	11678.47	9.40	–0.311	98.50	38.73	INSOLUBLE	0.70446
9	malS	17	11304.14	9.21	–0.297	93.73	44.85	INSOLUBLE	0.64645
10	spy	23	11878.72	9.63	–0.347	94.91	34.79	INSOLUBLE	0.73229
11	rseB	23	12011.84	9.40	–0.374	88.52	44.73	INSOLUBLE	0.57843
12	dsbC	20	11717.57	9.57	–0.384	84.57	37.84	INSOLUBLE	0.55781
13	amiA	34	13129.24	9.99	–0.414	91.01	49.41	INSOLUBLE	0.71947
14	fhuD	30	12887.97	9.89	–0.42	94.17	58.76	INSOLUBLE	0.61314
15	mglB	23	11900.80	9.57	–-0.356	89.44	38.82	INSOLUBLE	0.74340
16	eco	20	11648.45	9.40	–0.333	89.24	42.72	INSOLUBLE	0.81011
17	nikA	22	11972.89	9.51	–0.286	96.64	48.57	INSOLUBLE	0.51704
18	mepH	27	12507.56	9.51	–0.304	96.70	39.35	INSOLUBLE	0.85907
19	fadL	25	12191.04	9.57	–0.394	90.45	42.40	INSOLUBLE	0.60619
20	ompL	20	11645.49	9.57	–0.346	97.52	41.50	INSOLUBLE	0.75965
21	ompN	21	11597.49	9.57	–0.301	100.38	37.52	INSOLUBLE	0.57574
22	thiB	18	11512.50	9.26	–0.308	98.45	49.02	INSOLUBLE	0.75784

### In silico analysis of physicochemical characteristics of signal peptides

The physicochemical characteristics of PTH protein linked to the 32 signal peptides were evaluated based on the amino acid sequence using the ProtParam server. Structural and functional characteristics such as signal peptide length, molecular weight, PI, GRAVY, aliphatic index, and instability index were examined for the desired protein containing the studied signal peptides (Table 3).

**Table 3:** 

No.	Signal peptides	Aliphatic index	GRAVY	D-score	H-region length
1	ompN	100.38	–0.301	0.900	6–14(9)
2	appA	100.28	–0.275	0.787	5–16(12)
3	agp	98.50	–0.311	0.867	5–16(12)
4	ushA	98.45	–0.320	0.726	7–18(12)
5	thiB	98.45	–0.308	0.792	5–12(8)
6	ompL	97.52	–0.346	0.850	6–14(9)
7	dsbD	97.50	–0.282	0.625	5–13(9)
8	lptA	96.70	–0.326	0.899	12–21(10)
9	mepH	96.70	–0.304	0.788	10–21(12)
10	nikA	96.64	–0.286	0.621	8–16(9)
11	degQ	95.80	–0.346	0.742	7–21(15)
12	ptrA	95.74	–0.376	0.886	1–7(9)
13	spy	94.91	–0.347	0.776	6–17(12)
14	fhuD	94.17	–0.420	0.725	12–23(12)
15	malS	93.73	–0.297	0.805	4–11(8)
16	bepA	93.21	–0.335	0.759	9–20(12)
17	amiA	91.01	–0.414	0.661	19–27(9)
18	fadL	90.45	–0.394	0.773	10–19(10)
19	mglB	89.44	–0.356	0.860	5–16(12)
20	eco	89.24	–0.333	0.874	5–14(10)
21	rseB	88.52	–0.374	0.817	7–16(10)
22	dsbC	84.57	–0.384	0.850	5–13(9)

### Prediction of protein solubility

Nowadays, of the various servers applied to predict protein solubility, one of the most accurate and reliable servers is SOLpro. The solubility of the PTH in connection with various signal peptides and solubility percent for each structure was evaluated as shown in Table 3. The results showed that the PTH is insoluble when linked to all used signal peptides.

As a result, considering accomplished analysis to identify and rank the most appropriate signal peptides linked to PTH, 22 signal peptides were compared based on the mentioned parameters and then they were ordered using the aliphatic index. Finally, 12 signal peptides with an aliphatic index of more than 95 were selected for further analysis (Table 4).

**Table 4:** 

No.	Signal peptide	Cytoplasmic	Membrane	Secreted	Periplasmic	SecP score
1	ompN	0.00	0.54	0.03	9.43	0.36
2	appA	0.00	0.34	0.00	9.66	0.35
3	agp	0.00	0.00	0.18	9.82	0.42
4	ushA	0.16	0.00	0.00	9.84	0.33
5	thiB	0.00	0.38	0.04	9.59	0.34
6	ompL	0.00	0.10	9.61	0.29	0.32
7	dsbD	0.75	8.77	0.49	0.00	0.15
8	lptA	0.00	0.19	0.09	9.72	0.86
9	mepH	0.07	9.79	0.00	0.14	0.14
10	nikA	0.00	0.15	0.15	9.73	0.16
11	degQ	0.00	0.30	0.08	9.62	0.89
12	ptrA	0.00	0.08	0.20	9.72	0.43

### Prediction of the secretory protein location

Prediction of protein location is a vital step in drug designing. In this study, the ProtCompB server version 9 was employed to predict the final cellular location of PTH in *E. coli*. So, several logical algorithms were applied to determine different cellular location.

In this study, the secretory ability of the final signal peptides was investigated using the SecretomeP server. The final version of this server predicts the secretory ability of proteins by comparing their biochemical properties with cytoplasmic proteins. This server presents the prediction results as SecP score, and the threshold score for bacterial signal peptides is 0.5. Ten of the final signal peptides failed to obtain the minimum score for optimal PTH secretion and only two of them earned a score of more than 0.5. This score showed that these two final signal peptides have a high secretion potential rather than other signal peptides.

Based on the results of this study, which were obtained by evaluating several important parameters involved in the secretory expression, lptA and degQ signal peptides have greater potential for favorable secretory expression of PTH in bacterial systems.

## Discussion

Development of a new drug is a very complicated process that requires time and money. Nowadays, predictive methods are used as powerful and growingly important tools to save time and money in the process of new drugs development. Hence, several studies have used bioinformatics methods that increase the precision and accuracy of empirical studies, in addition to the above-mentioned benefits. Bioinformatics is an interdisciplinary field that is widely used in the processing of biological information through computational methods and making a link between different sciences such as computer and statistics [18, 19]. Overexpression of recombinant proteins in *E. coli* can lead to improper folding and accumulation of them in aggregated and insoluble intracellular bodies, named inclusion bodies [20, 21]. By adding a suitable signal peptide to the N-terminus of the desired protein, it can be directed to the periplasmic space and improper folding at the overexpression of recombinant proteins in *E. coli* can be resolved [22, 23]. To achieve an appropriate level of recombinant proteins secretion into the periplasmic space, the directed signal peptide of the desired protein should have proper features. In this regard, there are various computational tools to predict and identify a variety of physicochemical properties of a signal peptide, such as molecular weight, isoelectric pH, GRAVY, and aliphatic index.

The signal peptides selected for obtaining more levels of PTH secretion in *E. coli* were compared with each other. To achieve reliable results that can be generalized, it was avoided to use hypothetical signal peptides as far as possible. Appropriate signal peptides for the periplasmic expression of PTH gene were extracted from UniProtKB, one of the most reputable databases used in raw data extraction studies. Another reason for the use of UniProtKB is that it provides a set of functional data about proteins with accurate explanations and its users continuously collect, process, update, and display data in order to improve the availability and usability of these data [24, 25]. As a result, the access number of this reputable database was used for addressing the amino acid sequences of signal peptides.

Based on the results of the SignalP server, 10 signal peptides earned a D-score of lower than 0.5, which were excluded from the study because of the inability to be cleaved. In addition, 22 signal peptides exhibited the right cleavage site and proper cleavage potential that were used for further investigations.

Signal peptides are composed of three regions, namely H, N, and C. The N-terminus often contains positively-charged amino acids, H Region is the intermediate hydrophobic region that contains at least six amino acids, and the C-terminus consists of polarized uncharged amino acids and a signal peptide cleavage site. Given the impossibility of determining these three regions in the SignalP-4.1 server, the SignalP-3 was used to determine and study the triple areas in signal peptides (Tables 1 and 2). Although the triple regions of signal peptides do not function independently, the length of H Region is one of the most important parameters in protein secretion [26].

In an *in silico* study on 48 human signal peptides fused with the human growth hormone for periplasmic secretion, Douzandeh *et al*. also used the SignalP server to predict the signal peptides cleavage site and showed that only 17 signal peptides were eligible for further analysis [27].

The length of all signal peptides originated from gram-negative bacteria and ranged between 17 and 34 amino acids. Regularly, a signal peptide sequence in gram-negative bacteria contains 18-30 amino acids. Therefore, it can be stated that the studied signal peptides are in a proper range [28]. PI was calculated based on pKa values of amino acids. Since the pKa value of each amino acid depends on its side chain, PI plays a major role in determining the pH-dependent properties of a protein. In addition, protein GRAVY, the sum of hydropathy value of all amino acids divided by the length of the sequence, was calculated for all signal peptides.

Previous findings have shown that the increase in hydrophobicity levels and the length of H Region leads to the protein secretion improvement [29]. The aliphatic index indicates the hydrophobicity level of the studied sequence, which is defined as the relative volume occupied by the aliphatic side chains of alanine, leucine, isoleucine, and valine in the desired amino acid sequence. Calculated based on the sum of the mole fraction of above-mentioned amino acids, the aliphatic index can be regarded as a positive factor for the increase of thermostability of proteins, as it is significantly higher in thermophilic bacteria proteins than non-thermophilic ones[30]. The instability index, which estimates the stability of proteins in a normal state and in accordance with their initial structure, was also calculated for selected sequences (Table 3). Proteins with an instability index under and over 40 are predicted to be stable and unstable, respectively [31].

The hydrophobicity level of different signal peptides can be studied using parameters such as the aliphatic index, GRAVY, and the length of H Region. Therefore, signal peptides can be compared based on their hydrophobicity level by sorting the relevant data, besides another important index like the D-score of each sequence. In a study conducted by Douzandeh *et al*. on hGH, the same server was used to investigate various physicochemical characteristics of signal peptides.

Protein accumulation and folding in the cell environment are contradictory, as the overexpression of unfolded proteins leads to their accumulation and generation of inclusion bodies. Protein insolubility is a major barrier to the commercial production of protein-based pharmaceuticals [32].

Solubility is one of the major characteristics of every protein, which is directly associated with its amino acids sequences, and one of the important factors in the production, formulation, and preparation of pharmaceutical recombinant proteins, because subsequent measurements should be made on soluble proteins [33]. Intrinsic factors such as amino acids of the protein surface, along with non-intrinsic factors such as pH, ionic strength, and temperature, which can be measured in empirical observations, influence protein solubility [34]. With the increase in the use of proteins as pharmaceuticals and the need to express a large amount of them, the prediction of protein solubility has increasingly become important [33].

In most cases, the expression of heterologous proteins in bacteria leads to the generation of insoluble proteins and inclusion bodies [32]. The results of this stage were consistent with the findings obtained from similar studies in relation to the analysis of signal peptides fused with the human growth hormone (hGH), in which all signal peptides fused with hGH protein were predicted to be insoluble. For example, Douzandeh *et al*. used this server to evaluate the solubility of hGH protein in *E. coli*.

In a gram-negative bacterial host, the protein is predicted to be in one of the cytoplasmic, membrane (internal and external), secretory, and periplasmic areas. This server uses several methods to predict the protein placement, including neural network-based prediction, comparison to homologous proteins with a known location, and prediction of the desired peptide sequence performance.

The results indicated that 9 out of the 12 studied signal peptides have a great potential for PTH secretion into the periplasmic space, whereas 2 of them do not have the ability to cross the bacterial membrane and the last signal peptide secretes the protein out of the bacterium.

Ten of the final signal peptides failed to obtain the minimum score for optimal PTH secretion and only 2 of them earned a score over 0.5. This indicates the high secretion ability of these peptides compared to others.

## Conclusion

Empirical studies conducted on the periplasmic production of recombinant hormones in *E. coli* using a signal peptide derived from a gram-negative bacterium have reported different results in terms of efficiency. In addition, because of the need to spend a large amount of time and money, such studies are not cost-effective. Nowadays, several signal peptides fused with PTH can be investigated using bioinformatic studies, which both save time and cost and increase accuracy. The results of this study, obtained from several reputable servers in multiple stages, showed that signal peptides theoretically have the ability to periplasmically express this protein in *E. coli*. Moreover, based on the multistage evaluation of this study and the proven validity of bioinformatic surveys in the analysis of signal peptides, these results can be used empirically and also can influence other studies on the periplasmic expression of a variety of protein-based pharmaceuticals in *E. coli*. Based on the study findings, lptA and degQ signal peptides, in the case of fusion with human PTH, theoretically have greater potential for periplasmic expression than other studied signal peptides. Further studies in this area can lead to the confirmation of the results of the present research as well as the use of them in future empirical experiments.

## Conflict of Interest

The authors confirm that there are no conflicts of interest.
